# 1499. Tesamorelin Reduces Visceral Adipose Tissue and Liver Fat in INSTI-Treated Persons with HIV

**DOI:** 10.1093/ofid/ofad500.1334

**Published:** 2023-11-27

**Authors:** Taryn McLaughlin, Steven K Grinspoon, Takara Stanley, Lindsay Fourman

**Affiliations:** Theratechnologies, Inc, Seattle, Washington; Massachusetts General Hospital, Weston, Massachusetts; Massachusetts General Hospital, Weston, Massachusetts; Massachusetts General Hospital, Weston, Massachusetts

## Abstract

**Background:**

Significant attention surrounds the recent observation of weight gain in persons with HIV (PWH) initiating integrase inhibitors (INSTIs). Given the finding that visceral adipose tissue (VAT) is a critical component of this weight gain, the clinical implications of this weight gain are a growing concern, since VAT has been associated with downstream comorbidities including non-alcoholic fatty liver disease (NAFLD). Tesamorelin, a growth hormone releasing hormone analogue, has been shown to reduce VAT by over 15% in 6 months in PWH with lipohypertrophy. Therefore, we sought to investigate if tesamorelin could reduce VAT and liver fat in PWH on INSTI-containing regimens.

**Methods:**

To do this, we leveraged data from a previous placebo-controlled trial of 61 participants with HIV-associated NAFLD. Measurements of body composition and hepatic fat fraction (HFF) were taken at baseline and after 12 months of treatment. Post-hoc analyses were performed to evaluate changes in body composition amongst individuals receiving INSTIs.

**Results:**

Amongst participants, 39 (64%) were on INSTI containing regimens, the most common of which was dolutegravir (41%). These individuals were largely similar at baseline to those on regimens without INSTIs. Interestingly, although anthropometric measurements at baseline did not differ between the two groups, at 12 months, there were marked changes in VAT amongst the INSTI group. After 12 months, there was a significant difference in VAT change between the placebo and tesamorelin treated groups (p=0.0034). The placebo group had an increase in VAT of 10.8% whereas the Tesamorelin treated group had an overall reduction in VAT of 8.3%. Furthermore, the tesamorelin treated group saw a 31% relative reduction in HFF compared to baseline (p=0.006) which was significantly higher than in the placebo treated group (T:-4.9%, P:-0.1% absolute change p=0.015).

**Conclusion:**

These data reinforce the impact of INSTIs on VAT, body composition, and concomitant organs in PWH. It also demonstrates the potential for tesamorelin as a strategy to reduce VAT and HFF amongst individuals with INSTI-associated VAT accumulation.

**Disclosures:**

**Taryn McLaughlin, PhD**, Theratechnologies, Inc: Employee **Steven K. Grinspoon, MD**, Theratechnologies: Grant/Research Support **Takara Stanley, MD**, Pfizer, Inc: Grant/Research Support **Lindsay Fourman, MD**, Amryt Pharmaceuticals: Advisor/Consultant|Amryt Pharmaceuticals: Grant/Research Support

